# Membranes on the move: The functional role of the extracellular vesicle membrane for contact‐dependent cellular signalling

**DOI:** 10.1002/jev2.12436

**Published:** 2024-04-22

**Authors:** Kevin Jahnke, Oskar Staufer

**Affiliations:** ^1^ School of Engineering and Applied Sciences Harvard University Cambridge Massachusetts USA; ^2^ INM – Leibniz Institute for New Materials Saarbrücken Germany; ^3^ Helmholtz Institute for Pharmaceutical Research Saarbrücken Germany; ^4^ Center for Biophysics Saarland University Saarbrücken Germany; ^5^ Max Planck‐Bristol Center for Minimal Biology University of Bristol Bristol UK

**Keywords:** contact‐dependent signalling, extracellular vesicles, immunotherapy, membrane biology, signalosomes

## Abstract

Extracellular vesicles (EVs), lipid‐enclosed structures released by virtually all life forms, have gained significant attention due to their role in intercellular and interorganismal communication. Despite their recognized importance in disease processes and therapeutic applications, fundamental questions about their primary function remain. Here, we propose a different perspective on the primary function of EVs, arguing that they serve as essential elements providing membrane area for long‐distance, contact‐dependent cellular communication based on protein‐protein interaction. While EVs have been recognized as carriers of genetic information, additional unique advantages that they could provide for cellular communication remain unclear. Here, we introduce the concept that the substantial membrane area provided by EVs allows for membrane contact‐dependent interactions that could be central to their function. This membrane area enables the lateral diffusion and sorting of membrane ligands like proteins, polysaccharides or lipids in two dimensions, promoting avidity‐driven effects and assembly of co‐stimulatory architectures at the EV‐cell interface. The concept of vesicle‐induced receptor sequestration (VIRS), for example, describes how EVs confine and focus receptors at the EV contact site, promoting a dense local concentration of receptors into signalosomes. This process can increase the signalling strength of EV‐presented ligands by 10‐1000‐fold compared to their soluble counterparts. The speculations in this perspective advance our understanding of EV‐biology and have critical implications for EV‐based applications and therapeutics. We suggest a shift in perspective from viewing EVs merely as transporters of relevant nucleic acids and proteins to considering their unique biophysical properties as presentation platforms for long‐distance, contact‐dependent signalling. We therefore highlight the functional role of the EV membrane rather than their content. We further discuss how this signalling mechanism might be exploited by virus‐transformed or cancer cells to enhance immune‐evasive mechanisms.

## 1 INTRODUCTION

Extracellular vesicles (EVs) have gathered significant attention over the past two decades. These lipid‐enclosed structures are released by virtually all life forms across the tree of life and abundant in a biofluids from bacterial biofilms to human blood. They have been recognized as crucial for intercellular and even interorganismal communication (Sanwlani et al., 2020) and their role in diseases has been extensively studied, leading to the development of EV‐based therapies (Murphy et al., 2019; Yáñez‐Mó et al., 2015). Furthermore, our understanding of numerous biological processes has been significantly enhanced following their discovery (van Niel et al., 2018). However, fundamental questions about their biology, particularly their primary physiological functions, remain unanswered.

EVs are evidently a successful communication strategy, as they appeared early in primitive life forms (Askenase, 2021) and have maintained their basic structure across all kingdoms of life (Woith et al., 2019). But what is their unique advantage? What essential molecular benefits do they bring to living systems? As a structure so integral to life, the reasons for their existence should be deducible from first‐principle arguments. Contrary to the initial belief that EVs are merely waste products of cellular turnover and housekeeping, they have emerged as carriers of biomolecular content (Rashid et al., 2017) and numerous studies have reported on the ability of EVs to shuttle genetic and epigenetic regulatory elements between cells (Valadi et al., 2007). The EV lumen can transport nucleic acids and prevent their degradation, while providing cellular homing capabilities for directed transfer between cells. Although the lumen of most EVs is too small to carry large amounts of nucleic acids, smaller compounds such as microRNAs can be efficiently transported. Such regulatory molecules often already exert effects at low doses, wherefore transfer of only a few microRNA molecules to a target cell can already impact on its phenotype. Despite initial skepticism, this is now widely accepted as a fundamental function of EVs, at least in some communication processes in multicellular organisms as well as in microbial communities (Ñahui Palomino et al., 2021). While EVs provide a combination of key advantages for intercellular transfer of nucleic acids and other immunogenic or regulatory cargo, for example, targeting, shielding and intracellular transfer, nature has evolved several other strategies for such transfer. These include nucleic acid binding of proteins in serum (Thoburn et al., 1972), the emergence of viral capsids as DNA/RNA transporters, DNA‐binding polysaccharides (Limoli et al., 2015) or even free circulating nucleic acids (Kananen et al., 2023) which, at least in theory, could fulfil some of the functions provided by EVs.

**FIGURE 1:**
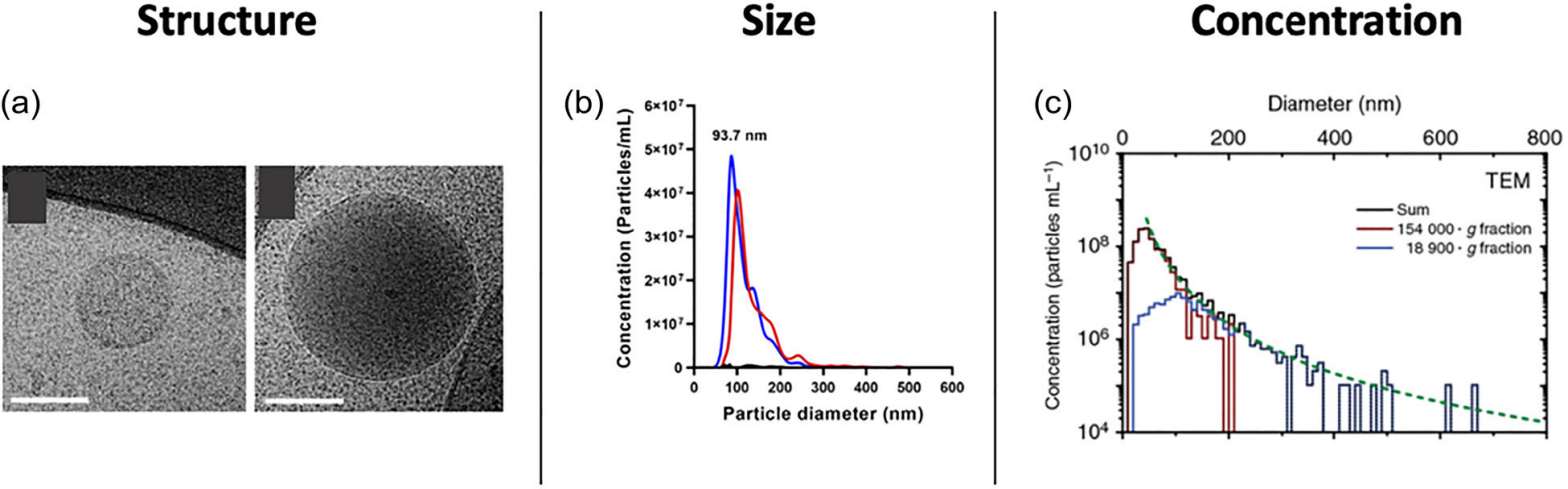
Typical EV structure, size and concentration. (a) Cryogenic transmission electron microscopy showing the spherical morphology of plasma‐derived EVs in solution. Reproduced and adapted with permission from Yuana et al. (2013). (b) Representative EV size distribution measured by nanoparticle tracking analysis from EV released in cell culture medium. Reproduced and adapted with permission from Contreras et al. (2023). (c) Urinary EV concentration as a function of size obtained by transmission electron microcopy. Reproduced and adapted with permission from van der Pol et al. (2014).

In larger multicellular organisms like humans, where EVs are prevalent in virtually all tissues and body fluids at high concentrations (Contreras et al., 2023), the majority of EVs are rather small in diameter and spherical in solution (Figure 1a). Most reports approximate the largest population of EVs to have diameters below 100 nm (van der Pol et al., 2014) (Figure 1b). Consequently, the volume of EVs available for transport is relatively small. For instance, in human body fluids, the concentration of EVs with a radius below 100 nm, is in the order of 10E10 to 10E11 EVs per millilitre (Johnsen et al., 2019; van der Pol et al., 2014) (Figure 1c). Considering all EVs in the blood, the total volume they provide and that is available for packing of DNA and RNA is only about 50 nL per millilitre (or 0.005% of the total volume).

However, despite their small size, EVs provide a substantial amount of membrane surface area. The membrane is a common feature shared by all EVs across all life forms (Gill et al., 2019), underscoring its fundamental importance to their function. While the size of serum EVs can range from 30 to 1000 nm in diameter (van der Pol et al., 2014), and their concentration can vary from millions to billions per millilitre depending on numerous factors, including an individual's health status, we can simplify by assuming an average diameter of 100 nm. This size corresponds to the largest peak in the EV size distribution range within most biofluids (van der Pol et al., 2014). With this assumption, and considering the vesicles as spherical, each EV provides a surface area of about 0.03 µm^2^. Assuming a concentration of 1E12 EVs in a millilitre of blood, the total membrane area provided by EVs in this volume would amount to approximately 0.031 m^2^. When compared to the membrane area provided by other cell types in 1 mL of blood (approximately 1 m^2^ for red blood cells, whose primary function is transport, and only 0.006 m^2^ for white blood cells and 0.023 m^2^ for platelets), EVs contribute about 3% of the total membrane area in blood an even provide more membrane are than white blood cells and platelets together, although these are rough estimates. Interestingly, from this estimate, EVs provide even more membrane area than leukocytes. This is noteworthy considering that the leukocyte cell membrane is a central structure, serving as the primary interface for detecting environmental signals and determining immunogenicity. Complex and highly sophisticated molecular machineries have evolved based on membranes, including structures like the immune synapse and the complement system, to provide specificity and effector functions to leukocytes. Membrane interfaces are especially crucial in the immune systems but also to most other cell functions (Belardi et al., 2020), just like EVs. Given the rough calculations above, it is plausible to suggest that the membrane area provided by EVs is central to their function. While the transport volume of EVs, particularly larger ones, is certainly not negligible, the amount of membrane area provided by these vesicles seems too significant to be overlooked. By studying and considering the role of the membrane provided by EVs, we could gain insights into the fundamental questions raised above and potentially uncover why they are a winning strategy for cellular communication.

In the following, we argue that EVs are essential elements to provide membrane area for long distance contact‐dependent cellular communication based on protein‐protein interaction and other membrane‐ligands. A membrane is essential to this process as it provides the uniqueness of 2D‐restricted diffusion that can lead to ligand clustering.

### 1.1 The four pillars of intercellular communication

**FIGURE 2:**
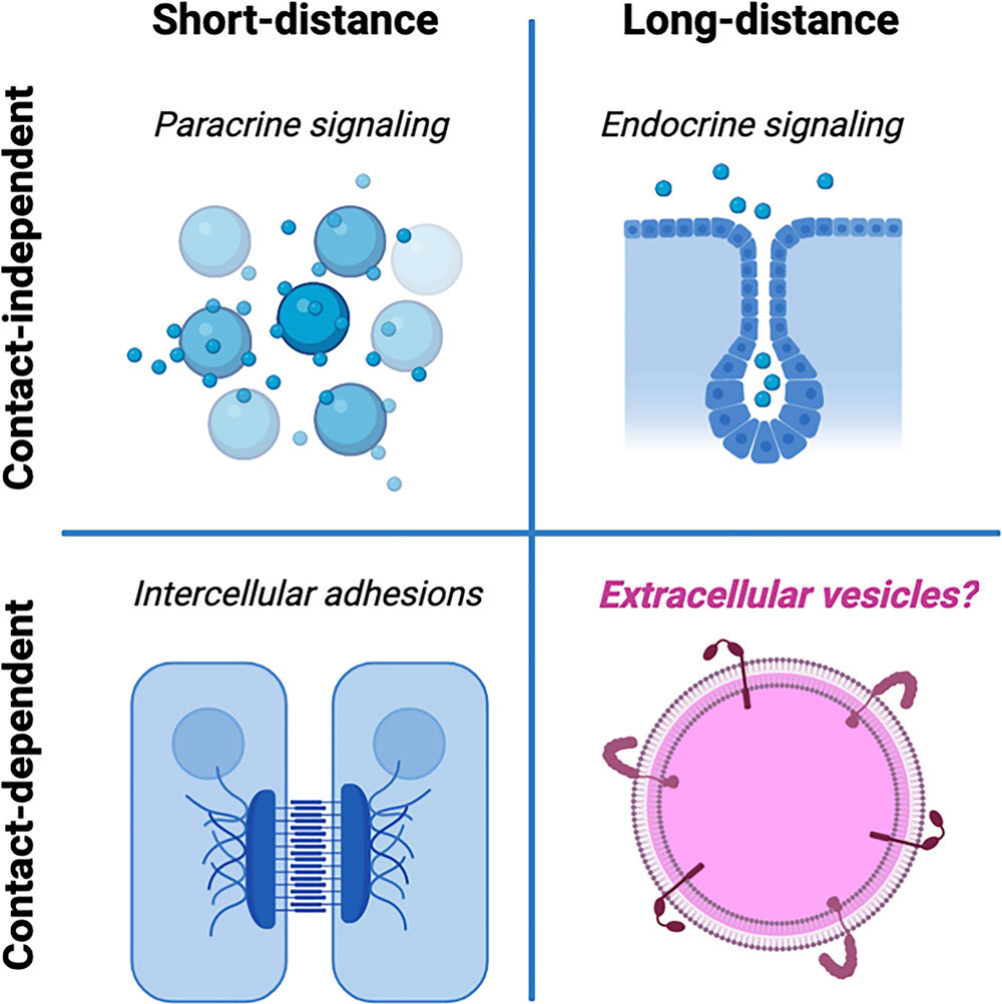
The four pillars of intercellular communication along the two axes of long/short distance and contact independent/dependent signalling.

Cells have developed a multitude of communication pathways, each uniquely adapted to the requirements of various physiological processes. Within animals, such intercellular communication mechanisms need to span several orders of magnitude in both time and space. On the temporal scale, these mechanisms range from rapid signal transduction at neuronal synapses (Faber & Pereda, 2018), which occurs in milliseconds, to slower processes such as mechano signalling during wound healing, which can take several days (Das et al., 2015). Spatially, these communication pathways can range from direct cell‐to‐cell signalling via cadherins (nanometer range) (Campbell et al., 2017), to long‐distance communication across complex systems like the gut‐brain axis (meter range) (Rutsch et al., 2020). In essence, the complexity and diversity of cellular communication reflect the intricate and multifaceted nature of life itself and can be classified across two axes: contact‐dependent and contact‐independent as well as long‐distance and short‐distance communication (Figure 2).

1. **Contact‐independent, short‐distance signalling**: This category includes mechanisms like paracrine signalling, where cells release signalling molecules such as growth factors (e.g., VEGF (Villegas et al., 2005)) that act on nearby cells and extracellular calcium waves, which play a crucial role in processes like bone remodelling (Jørgensen, 2005).
2. **Contact‐independent, long‐distance signalling**: The most well‐studied example in this category is endocrine signalling, where hormones are secreted from one organ (e.g., insulin in the pancreas) and travel through the bloodstream to target distant tissues to regulate glucose levels.
3. **Contact‐dependent, short‐distance signalling**: This category encompasses all intercellular adhesion axes, including those involved in epithelial mechanotransduction (Leckband & de Rooij, 2014). It also includes receptor‐ligand interactions, such as the Notch‐Delta signalling pathway (Kopan, 2012), where direct contact between cells is required for signal transmission, or tunnelling nanotubes for direct transfer of cytoplasmatic components (Rustom et al., 2004).
4. **Contact‐dependent, long‐distance signalling**: Traditional examples in this category are less common. In some animals, neurons exemplify this type of signalling, as they extend long projections throughout the body to make direct contact for long‐distance interactions. However, this is a unique case. EVs could represent a prevalent and universal type of signalling mechanism in this category, as they can be released by one cell and make direct contact with a distant target cell to transmit signals.

The substantial membrane area provided by EVs, allowing for contact dependent interactions, suggests that they could effectively fill the gap in the fourth category, potentially serving as a highly efficient, and universally applicable signalling mechanism to facilitate long‐distance, contact‐dependent signalling. But what specific advantage do EVs offer compared to other diffusive particle types or even more complex cellular architectures (e.g., axons and other cell projections)? Conceptually, the first two types of communication axes are driven by the passive diffusion of signalling compounds, making this a low energy demanding process. However, it lacks directionality, making it challenging to control directed signalling to ensure that information specifically and effectively arrives at the intended receiving cells. In contrast, EVs, while also lacking an intrinsic mechanism for active transport and primarily moved by passive diffusion, can be directed to specific cells. Proteins and carbohydrates on their membrane can guide EVs to specific tissues (Edelmann & Kima, 2022), thereby combining the unique advantages of passive, low‐energy transport and directed signalling. Long cellular projections in turn, are typically static, displaying limited plasticity over short time intervals. They require significant energy to construct and sustain. Consequently, their remodelling (e.g., axons and dendrites in the brain) is stringently regulated and tends to be slow‐paced. Furthermore, these projections are highly directional, often targeting only a limited number of cells simultaneously, which restricts their effectiveness for broad communication. However, these considerations do not explain why a lipid membrane is necessary and why other particles and aggregates, such as lipoprotein‐like structures, have not emerged as a winning strategy. What makes the lipid membrane instrumental for the functioning of EVs in contact‐dependent long‐distance communication?

### 1.2 The EV membrane as protein presentation platform

**FIGURE 3:**
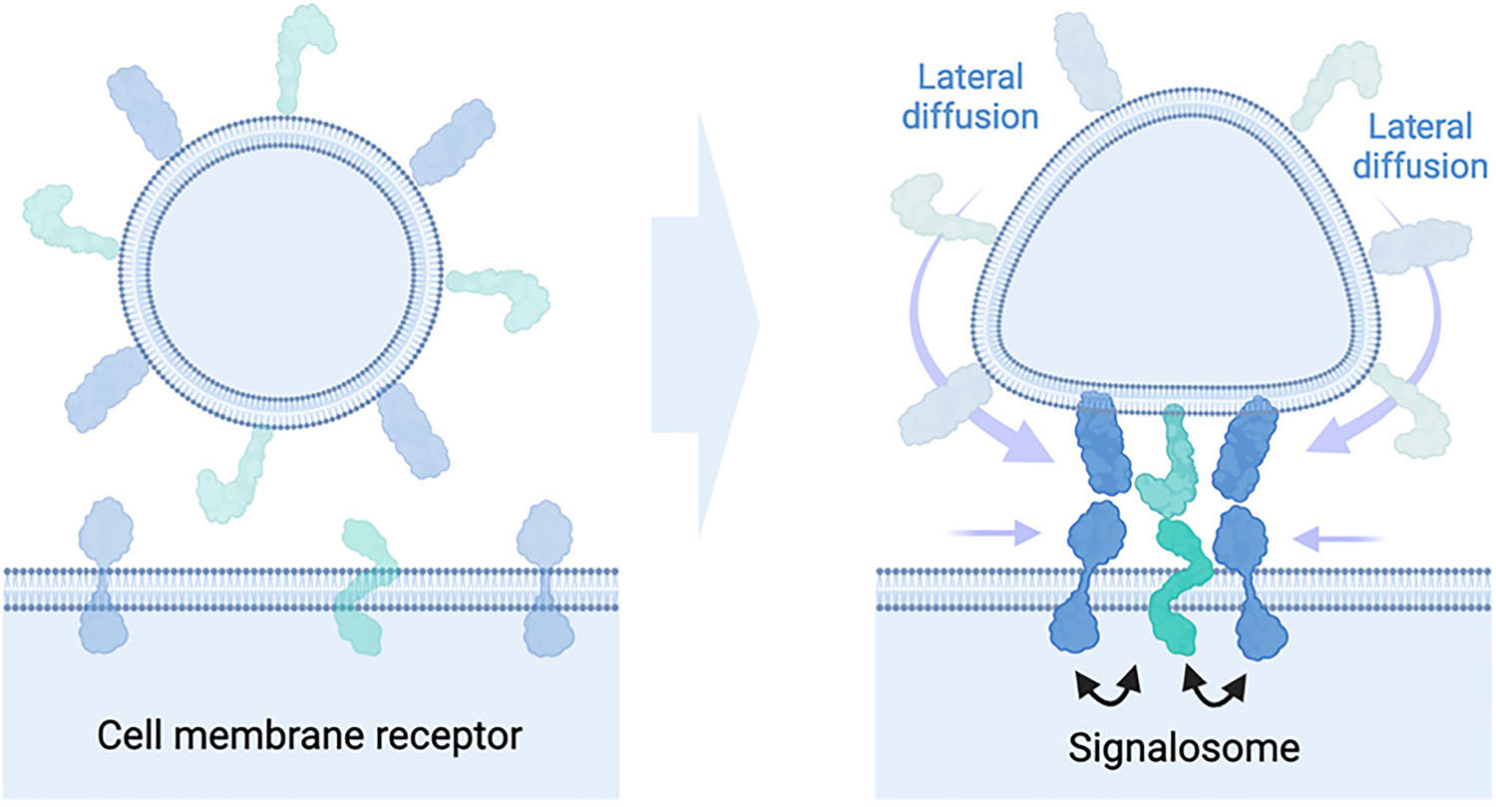
Vesicle‐induced receptor sequestration at the EV‐cell membrane interface. Aggregation of receptors at the EV contact site drives signalosome assembly.

Membranes are not only the central barriers that define cellular entities, but they are also the most essential platforms to enable, support and regulate receptor‐ligand interactions (Belardi et al., 2020). Cell membrane receptors, including receptor tyrosine kinases, the TNF‐receptor superfamily, and integrins, are crucial for directing the flow of information into cells (Westerfield & Barrera, 2020; Zhang et al., 2021). Upon activation, these receptors cluster with other receptors and co‐stimulatory molecules, forming larger signalosome structures like focal adhesions or supramolecular activation clusters in the immune synapse (Burridge, 2017; Dustin, 2014). This allows for precise signalling, increased sensitivity, and long‐lasting interactions. EVs can play a significant role in this process as they provide membrane area and present exofacial receptor ligands on their surface (Buzás et al., 2018). When an EV contacts a cell, the ligands on the EV membrane can diffuse to this contact zone and concentrate there due to their binding to their cognate receptors (Figure 3). This confines and focuses the receptors locally at the EV‐cell contact site (depending on the EV size only some nm^2^ in diameter) and facilitates the formation of receptor patches (Morandi et al., 2022). This process, that we previously described as vesicle‐induced receptor sequestration (VIRS) (Staufer et al., 2022), promotes a dense local concentration of receptors and their interactions, thereby promoting multimerization and downstream signalling. The VIRS effect can increase the signalling strength of ligands 10‐1000‐fold compared to their soluble counterparts. VIRS‐driven effects have been quantified in several experimental systems and through multiscale simulations for different ligand classes down to the differential regulation of gene transcription (e.g., IL‐12, TNF alpha or CD40L (Elmetwali et al., 2020; Su et al., 2022; Zhang et al., 2023)). Could one of the EVs main functions be to enable and sustain receptor‐ligand interactions as membrane platforms?

Supporting this notion, we can consider the receptor ligands presented on the surfaces of EVs. Hundreds of such ligands have been reported, and interestingly, many of these are not exclusive to EV surfaces but also exist in a soluble form. This includes several ligands for major receptor families, such as the TNF family receptor ligands (e.g., CD95L (FasL) (Audo et al., 2012; Kim et al., 2005), TRAIL (Wajant, 2006), TNF alpha (Zhang et al., 2006), CD40L (Martínez et al., 2022), OX40L (Neyrinck‐Leglantier et al., 2022), CD70 (Himbert et al., 2020), CD30L (Hansen et al., 2014, 2016) and RANKL (Wnt (Gross et al., 2012; Holliday et al., 2021))) and Wnt ligands (Torres et al., 2021) as well as ligands belonging to the Ig superfamily (e.g., PD‐L1 (Martínez et al., 2022), CD80 (Liu et al., 2023), and CD66a/CEACAM1 (Igami et al., 2022)). Additionally, ligands for receptor tyrosine kinases such as VEGF (Zeng & Fu, 2019), colony‐stimulating factors (Meng et al., 2021), and epidermal growth factors (Frawley & Piskareva, 2020) have been identified on EVs. All these ligands have been detected in both EV‐bound and soluble forms. However, comparative studies for most of these ligands have demonstrated that the EV‐bound form exhibits much stronger signalling potencies compared to the soluble form (Bailly et al., 2021; Elmetwali et al., 2010; Haile et al., 2014; Lahmar et al., 2008; Li et al., 1999; Matsumoto et al., 2015; Richter et al., 2012; Suda et al., 1997). This is likely due to the VIRS effect, which can potentiate the local accumulation of the corresponding receptors, thereby enhancing their signalling capacity.

In this view, a membrane enclosed particle is crucial, as only membranes allow for the lateral diffusion and sorting of membrane ligands in two dimensions. This further promotes avidity‐driven effects and assembly of co‐stimulatory architectures at the EV‐cell interface. It also facilitates presentation of multiple co‐signalling ligands in a small place in space and maybe even more importantly, at the same time. Timing is a central factor for signal propagation and the timing of ligand presentation can determine cell fate (Khan et al., 2015). With this, EVs could further support the timing‐aspect of multifactorial signalling and the assembly of heterotypic signalosome structures. An EV‐supported signalosome could not only be relevant for different physiological signalling axes in humans or animals but most likely pertain to all forms of multicellular organisms that need to coordinate their specialized cellular architectures as well as multicellular communities in micro‐ecosystems. In all of this, the EV membrane serves as the core structural element and organization unit, that restricts the diffusion of ligands to a defined 2D plane, while the compartment as a whole drives the directionality of communication towards specific cell types and ensures simultaneous presentation of ligands.

Of note, this concept is not only pertinent to the fundamental progression in our understanding of biology, but it also holds critical implications for EV‐based applications and therapeutics. As several current approaches aim to present therapeutic proteinogenic agents on EVs (e.g., TNF‐family proteins and antibodies (Cheng et al., 2020; Yuan et al., 2017)), the VIRS effect and its amplifying nature must be taken into account from a pharmacodynamic perspective. Moreover, understanding EV‐based signalling mechanisms under pathophysiological conditions will require a shift in perspective. In addition to viewing EVs as transporters of relevant soluble proteins and genetic elements, we should consider their role in presenting membrane‐bound proteins to other cells in long‐distance, contact‐dependent signalling axes. In the broader context of understanding disease mechanisms, future research could explore how virus‐transformed or cancer cells might exploit this effect to enhance immune‐evasive mechanisms. These cells could potentially harness the membrane presentation abilities of EVs to potentiate the reprogramming of cells in their microenvironment.

### 1.3 Predictions and initial evidence

Considering the proposed model of a membrane‐centred functional role in EV signalling, several predictions arise, for which initial evidence can be gathered from existing data and biophysical considerations.

*Prediction 1: Single EVs present enough protein ligands on their surface to induce local clustering of multiple receptors*.

Receptor di‐ or trimerization is often deemed sufficient to initiate downstream signalling for many receptor classes. However, most outside‐in signalling events involve the formation of larger signalosome assemblies or the creation of specialized architectural patches of receptors that organize numerous receptors and co‐regulatory proteins. While certain receptor systems, like G protein‐coupled receptors that are coupled to signal amplification mechanisms, can be activated by a single ligand, the majority of receptor types require the simultaneous activation and multimerization of multiple receptors to effectively transduce signals into the cell. Typically, this involves activating a range of two to several hundred receptors on the cell surface, depending on the signalling threshold, the amplification mechanism, the level of pathway redundancy and feedback control (Huang et al., 2016; Ng et al., 2013; Schodin et al., 1996; Stubb et al., 2019). Correspondingly, a single EV would need to carry multiple receptor ligands to initiate multimerization. Assuming the ligand diffusion coefficient to be on the order of 1 µm^2^/s, which is similar to a lipid diffusion coefficient, and an EV diameter of 100 nm, it would take about seconds or less for one ligand to reach a second ligand at the EV‐cell interface that is already bound to its cognate receptor. From previous modelling of receptor multimerization based on the VIRS effect, first multimerization events are expected already with the first second after vesicle contact formation. Quantification of the protein density on EVs has remained challenging but first studies based on quantitative single EV flow cytometry have demonstrated that a single EV can carry multiple copies of a receptor ligand on the surface (e.g., CD45 or CD31) (Görgens et al., 2019; Woud et al., 2022). If this is the case for all major functional ligands identified on EVs so far, remains to be addressed experimentally.

*Prediction 2: EVs can simultaneously present multiple ligands on their surface*.

Related to the first prediction, our model suggests that single EVs are equipped with a diverse array of receptor ligands, each integral for a fine‐tuned modulation of cellular signalling processes. Single receptor activation is often insufficient for coordinated signalling. For instance, in the context of T lymphocyte activation, it is not merely the engagement of the T cell receptor that is pivotal; the concurrent activation of co‐stimulatory receptors (e.g., CD28), is indispensable. Predominantly, cellular processes are governed by a coordinated interplay of activations, encompassing both co‐stimulatory and inhibitory ligands, to achieve precise cellular regulation. While this might not be an absolute necessity for primary and regulatory ligands to coexist on the same vesicle for effective modulation, the proposed model postulates their co‐localization on a singular vesicle. Such an arrangement would be conducive to the assembly of localized signalosomes. While there is a plethora of experimental data underscoring that EV populations transport multitude ligands simultaneously, definitive empirical evidence elucidating the co‐presentation of several interrelated receptor ligands on an individual EV remains elusive.

*Prediction 3: Cells produce enough EVs to ensure efficient long‐distance signalling*.

To confer efficient signalling, enough EVs would need to be released to reach target cells in an appropriate time. Connected to this requirement is the question: What is a long‐distance in the context of EV‐based communication? Several reports show that EVs can act over extremely short and long distances. For example, T cell receptor‐enriched EVs have been observed in the nanometre‐wide synaptic cleft between antigen‐presenting cells and T cells (Céspedes et al., 2022). Conversely, cancer cell‐released EVs, when introduced into the bloodstream, have been found in metastatic niches within other organs (Guo et al., 2019). At first, EV transport within a tissue relies on passive transport, implying their effective radius from the source cell is relatively small during physiological time frames (hours to days). Neglecting limited diffusion in the dense extracellular space, an EV with a 100 nm diameter diffusing in solution over 24 h would have a root mean square displacement of more than 300 µm, equivalent to 30 cell diameters. Compared to liposome diffusion measurements (Pluen et al., 2001; Wang et al., 2012), the actual mean square displacement is expected to be at least an order of magnitude lower in the dense extracellular space. Therefore, the ‘passive’ communication radius of EVs is constrained and potentially also mediated by cellular projections such as tunnelling nanotubes or filopodia. However, tissues experience interstitial fluid flows, continuous lymph drainage, and pressure gradients, which could actively and directionally transport EVs. Consequently, the communication radius of EVs may not be limited to neighbouring cells, but can potentially establish efficient communication over several millimetres, even without considering more complex transport routes like lymph and blood vessels. This could be an effective intra‐tissue communication axis (scale of mm) where physiological responses need to be coordinated in space and time. A well‐studied and probably representative example for such a communication axis is the EV‐based transport of RANK and RANKL between osteoblasts and osteoclasts during bone remodelling (Holliday et al., 2021). Based on the microscale anatomy of the endosteal niche, this process needs to be coordinated across length scales of 0.1–1 mm (Lévesque et al., 2010). Given that individual cells can release several hundred EVs per hour (Chiu et al., 2016) and even a single EV can activate contact‐dependent signalling in its recipient cell (Sung et al., 2020), this range is adequately addressed by the signalling capacity of EVs.

*Prediction 4: Proteins can move laterally within the EV membrane*.

An integral prediction of the proposed model is that ligands in the EV membrane are laterally mobile. However, direct measurement of ligand mobility and extraction of diffusion coefficients within the membrane of single isolated EVs is technically very challenging. The use of solvent sensitive membrane probes that allow to deduce the lipid ordering state of EV membranes have proven helpful in this direction. Several studies have demonstrated that EV membranes are in a liquid ordered state and therefore laterally mobile (Suga et al., 2021; Yasuda et al., 2022). While this is no direct proof that EV membrane‐embedded proteins are mobile as well, as cytoskeletal elements within the EVs could restrict their diffusion, it suggests that they display some sort of mobility in the relevant physiological time frames. Future studies, potentially based on super resolution microscopy tracking of individual proteins in EV membranes, will need to quantify protein mobility on EV membranes.

*Prediction 5: EVs stay attached to cell membranes long enough to facilitate signalosome assembly prior to internalization or fusion*.

The interactions between EVs and target cell membranes have mostly been analysed by focusing on EV fusion and uptake. However, a critical prediction and requirement for our model is that EVs reside on cell membranes in an attached state long enough to allow for signalosome assembly. The formation of stable receptor‐ligand interactions and the assembly of receptor clusters is a highly dynamic process that typically requires seconds to minutes. So far there is only limited experimental data on the time that EVs reside on cell membranes under physiological conditions. Initial single EV tracking data in combination with optical tweezer positioning of EVs on cell membranes have shown that EVs can remain attached on cells for several seconds to minutes and even surf on their membrane before uptake and fusion (Heusermann et al., 2016; Prada & Meldolesi, 2016). This opens a suitable time window in which a VIRS effect could unfold. However, there is a lack of data that would support this notion under in vivo conditions.

*Prediction 6: When attached to a membrane, EVs offer a sizable membrane area, possibly through deformation, suitable for signalosome assembly*.

Larger signalosome assemblies, such as T cell receptor microclusters or focal adhesion sites require a minimal size to be functional and able to induce signalling. Typically, they feature sizes in between 10–400 nm (diameter), which is within the range of the membrane area provided by single EVs (Crescitelli et al., 2021; Horzum et al., 2014; Kandy et al., 2021; Yi et al., 2019). The contact area between an EV and a target cell membrane needs to be large enough to allow for receptor‐ligand interactions and the formation of a sufficiently large signalosome. Although, qualitative electron microscopy imaging data exist that show the deformation of EVs on cell membranes from a spherical shaped vesicle as well as the formation of a larger interaction plane between the two membranes (Simeone et al., 2020), no systematic and quantitative analysis for such an effect has been reported. EVs can be readily imaged when in contact with the cell membrane. However, it is difficult to discriminate if these vesicles are in the process of cellular attachment and up to be endocytosed or if they have just been released from the cell. However, they demonstrate that EVs are deformable particles and that a planar interaction interface between the EV and the cell membrane can be formed (Bigagli et al., 2016; Edgar et al., 2016; Khasabova et al., 2023; Salas et al., 2021). Given that a single EV could engage in 5–50 ligand–receptor interactions on a cell membrane with a typical binding energy of approximately 10 kJ/mol per receptor (which equals 4 k_B_T at room temperature) and assuming a membrane bending rigidity close to synthetic lipid membranes of 20–30 k_B_T (Faizi et al., 2022), already a few ligand‐receptor interactions can be sufficient to induce EV deformation.

## 2 CONCLUSIONS

In their essence, EVs provide a membrane‐to‐membrane interface that enables lateral diffusion of ligands. Given the large membrane area that EVs present to cells, they emerge as a pivotal element in the four‐pillar model of intercellular communication. By serving as membrane presentation platforms, the EVs main function could be to mediate and potentiate long‐distance, contact‐dependent communication based on protein, lipid or polysaccharide interactions. This does not negate the view that EVs can also act as transporters for (epi)genetic elements, for which considerable experimental proof has been provided, specifically in the context of biomedical applications involving exogenous injection of EVs. However, it does emphasize the membrane's functional role, which extends beyond mere protection of cargo.

## AUTHOR CONTRIBUTIONS

**Kevin Jahnke**: Methodology (equal); writing—original draft (equal); writing—review and editing (equal). **Oskar Staufer**: Conceptualization (equal); writing—original draft (equal); writing—review and editing (equal).

## CONFLICT OF INTEREST STAEMENT

The authors declare no conflict of interest.
